# The association between polychlorinated dibenzo-p-dioxin exposure and cancer mortality in the general population: a cohort study

**DOI:** 10.3389/fpubh.2024.1354149

**Published:** 2024-02-12

**Authors:** Lei Zheng, Xianli Zhang, Zhe Gao, Chunyu Zhong, Dezhi Qiu, Qing Yan

**Affiliations:** Department of Neurosurgery, Children’s Hospital of Nanjing Medical University, Nanjing, China

**Keywords:** polychlorinated dibenzo-p-dioxins, NHANES, cancer mortality, general population, restricted cubic spline (RCS)

## Abstract

**Introduction:**

Earlier research has indicated that being exposed to polychlorinated dibenzo-p-dioxins (PCDDs) in the workplace can heighten the likelihood of cancer-related deaths. Nevertheless, there is limited information available regarding the connection between PCDD exposure and the risk of cancer mortality in the general population (i.e., individuals not exposed to these substances through their occupation).

**Methods:**

The National Health and Nutrition Examination Survey (NHANES) detected PCDDs in the general population, and the death data were recently updated as of December 31, 2019. We conducted Cox regression analysis and controlled for covariates including age, gender, ethnicity, educational attainment, physical activity, alcohol intake, NHANES survey period, BMI category, cotinine concentration, and household earnings.

**Results:**

After accounting for confounding factors, the findings indicated that for each incremental rise of 1 log unit in 1,2,3,4,6,7,8,9-octachlorodibenzo-p-dioxin, there was a 76% rise in the likelihood of death from any cause, with a *p* value of 0.003. An increase of 1 log unit in the concentration of 1,2,3,4,6,7,8-heptachlorodibenzofuran could potentially lead to a 90% higher risk of cancer mortality, as indicated by a *p* value of 0.034 and a 95% confidence interval of 0.05–2.43. As the concentrations of 1,2,3,4,6,7,8-heptachlorodibenzofuran increased, the dose–response curve indicated a proportional rise in the risk of cancer mortality, accompanied by a linear *p* value of 0.044. The sensitivity analysis demonstrated that our findings were resilient.

**Discussion:**

In the general population, an elevated risk of cancer mortality was observed in PCDDs due to the presence of 1,2,3,4,6,7,8-heptachlorodibenzofuran. Mechanistic research is required to further confirm it.

## Introduction

PCDDs, also known as polychlorinated dibenzo-p-dioxins, are aromatic compounds that are widely found in the environment and are difficult to degrade. PCDDs can originate from various sources, such as incineration facilities for solid waste, mills involved in pulp and paper production, fish that have been contaminated, and soil. The accumulation of PCDDs in the human body can occur through the food chain and biological enrichment, resulting in detrimental impacts on human health due to their extended half-life. Earlier research has indicated that PCDDs can lead to a range of adverse health effects in humans, such as reproductive harm (1), hypertension symptoms (2), coronary heart disease, diabetes, nervous system impairment, disruption of endocrine, immune, and reproductive systems (3), and the development of tumors (4, 5). Nevertheless, limited research has been conducted on the correlation between PCDDs and the mortality rate of cancer.

According to previous studies, occupational exposure to PCDDs can heighten the likelihood of dying from cancer (6–9). According to Li et al. (10), among workers of a municipal solid waste incinerator plant the level of PCDDs in serum due to occupational exposure is 28.0 pg/g lipid. Compared with occupational exposure, non occupational exposure has a lower level of PCDD. Nevertheless, the concentration of serum PCDDs in individuals not occupationally exposed (i.e., the general population) is 8.0 pg/g lipid for males and 9.0 pg/g lipid for females, as reported by Chen et al. in 2005. Whether relatively low PCDD exposure level can increase the risk of cancer mortality needs more research evidence. In a previous study, it was found that PCDDs were linked to an increased risk of cancer death over a period of 4.63 years. The hazard ratio (HR) and its corresponding 95% confidence interval were reported as 1.17 (0.89–1.53), but these findings did not reach statistical significance (11). The short duration of the follow-up period could be the cause of these insignificant findings. Hence, further investigation with long follow-up time is required to determine the of PCDDs non-exposure among the general population and its association with cancer mortality.

In this research, an extended period of observation was utilized to investigate the correlation between PCDDs and the death rate caused by cancer. We examine the correlation between the collective exposure levels of PCDDs and individual PCDD exposure levels, as well as the risk of cancer mortality, by employing a combination of statistical techniques that incorporate exposure models and single exposure models.

## Methods

### Study participants

The NHANES is a survey conducted by the NCHS that aims to be representative of the entire nation, focusing on health and nutrition. In this study, we used a sample of adults collected from three survey cycles of NHANES from 1999 to 2004 to investigate the association between PCDDs exposures and cancer mortality. More details can be found on the NHANES official website below.^1^ The inclusion criteria included subjects who underwent PCDDs measurement and those who had mortality data (*N* = 3,497). The exclusion criteria was participant who were less than 40 years old (*N* = 505). Ultimately, 2,992 individuals were included in our analysis. A Cox proportional hazards model was generated to estimate the association of PCDDs and genetic factors with all-cause and cancer mortality and to assess the hazard ratio (HR) and 95% confidence interval (CI) with covariants. The restricted cubic spline (RCS) method was performed to investigate the association between PCDDs and cancer mortality. We performed sensitivity analysis guarantees the reliability of the findings. The survey was authorized by the NCHS Research Ethics Review Board. All study participants submitted written informed consent.

### Serum PCDD detection

High-resolution mass spectrometry (HRMS) was used to detect PCDDs in serum through high-resolution gas chromatography and isotope dilution (ID-HRMS). The specific methods are as follows. The serum samples were supplemented with the internal standard, and then the PCDDs were separated using either C18 solid-phase extraction or liquid–liquid extraction methods. Subsequently, multicolumn automatic purification and enrichment procedures were employed. A Hewlett-Packard 6,890 gas chromatograph was utilized to chromatograph the PCDDs, and the quantification of the chosen PCDDs was done through ID-HRMS using selective ion monitoring (SIM) and in EI mode using either a Micromass AutoSpec ULTIMA or Finnigan MAT95 mass spectrometer. To ensure quality control, the following procedure is implemented: each PCDD concentration is calibrated and calculated using an individual standard curve. Each detection and analysis run was carried out by a blinded method. The makeup of the examined samples was not known, and for detection, the method blank, quality control samples, and serum samples to be tested were utilized. Simultaneously, the overall mass and lipids of every sample were measured and adjusted to determine the PCDD concentration following lipid correction. Table 1 displays the detection rates of the PCDDs, represented as medians and quartiles. Because the distributions of the PCDDs were all skewed, these data were all log 10-transformed in the subsequent statistical analysis.

**Table 1:** The medians and quartiles and the detection rates of PCDDs.

PCDDs (pg/g)	>LOD (%)	25%	Median	75%
1,2,3,6,7,8-Hexachlorodibenzo-p-dioxin	84.1	3.6	32.4	57.5
1,2,3,4,6,7,8-Heptachlororodibenzo-p-dioxin	93.4	12.6	36.9	69.5
1,2,3,4,6,7,8,9-Octachlorodibenzo-p-dioxin	91.7	95.4	289	554
1,2,3,4,6,7,8-Heptachlorodibenzofuran	80.3	1.7	6.1	10.4
3,3′,4,4′,5-Pentachlorobiphenyl	87.7	4.9	21.7	45.3

PCDD, polychlorinated dibenzo-p-dioxin. LOD, limit of detection.

### Outcome definition

We obtained relevant mortality data from the NCHS, which can match the study subjects of the public database of consecutive NHANES from 1999 to 2004. These documents provide survey registry mortality data updated as of December 31, 2019. Data on deaths related to NCHS include information on the origin and reason for the deceased’s passing. The guidelines for all causes of death adhered to the 10th edition of the International Classification of Statistics, Diseases, Injuries, and Causes of Death (ICD-10). In accordance with the 10th edition of the International Classification of Diseases (ICD-10), we established the definitions for mortality from any cause and mortality specifically related to cancer (codes c00-c97), based on a prior investigation conducted by Duan et al. (12).

### Covariates

To adjust the results of the Cox regression and RCS analyses, the provided baseline information was utilized as a covariate. The variables that are considered are age, gender, ethnicity, body mass index (BMI) category, and NHANES period. Some variables considered to be possibly related to cancer mortality were also included in covariates, including alcohol consumption, serum cotinine (reflecting smoking), and activity level. Questionnaires are used to gather the aforementioned details, including but not limited to age, gender, race, education level, poverty income ratio (PIR), alcohol consumption, and activity level. BMI levels were calculated by measuring height and weight. Serum cotinine is obtained by detecting cotinine in serum. The intensity of physical activity was obtained through a questionnaire survey and was divided into three categories: none, moderate, and vigorous. Drinking alcohol status was based on the following question: “Have you had at least 12 drinks of any type of alcoholic beverage?” We divided the answers into “No” and “Yes.”

### Statistical analysis

When the data were continuous variables, the baseline information was presented as the average ± deviation from the norm. Percentages are used to present categorical variables. For continuous variables, if they were normality, the *t*-test were used; if they do not conform to normality, the Wilcoxon test were used. For categorical variables, chi-square test was used. We used Cox proportional hazards models with time in study as the underlying time metric to calculate the hazard ratios and corresponding 95% confidence intervals for cancer mortality in relation to blood PCDDs concentrations. We examined the proportional hazards assumption by creating a cross product of follow-up time and PCDDs levels. Likelihood ratio tests comparing models with and without this variable were not significant, suggesting no departure from the proportional hazards assumption. Continuous PCDD refers to the increased risk of all-cause and cancer mortality for each additional unit of PCDD. The RCS method was used to examine the correlation between PCDDs and cancer mortality in a dose–response relationship. Restricted cubic spline analysis with 3 knots (10th, 50th, and 90th percentiles) was used to examine the nonlinear association of blood PCDDs levels with cancer mortality within the values between the first and 95th percentile to minimize the influence of potential outliers. Nonlinearity was tested using the likelihood ratio test. To ensure the reliability of the findings, sensitivity analyses were performed. One analysis is to remove the PIR variable in the model. The other is to add educational status as a variable in the model. R software (version 4.04) was used to conduct all statistical analyses in this study. Statistical significance was determined if the *p* value, which was less than 0.05, indicated a two-sided outcome.

## Results

The study included a total of 2,992 participants, comprising of 1,461 males and 1,531 females (Table 2). The mean follow-up time was 138.3 months. In total, 1,002 people died, of whom 230 died from cancer. The racial distribution did not differ between males and females (*p* = 0.835). In women, the occurrence rate of PIR below 1 was greater compared to men, with a *p*-value less than 0.001. Men had a higher level of smoking exposure, with 36.5% of them having a cotinine frequency greater than 10, compared to 23.5% in women. BMI classification between men and women also has a different distribution. The male vigorous group accounted for 52.9% more than the female group, which had a significant difference (*p* < 0.001), among the exercise variables. The proportion of males in the drinking group was significantly higher than that of females, with 57.4 and 40.6%, respectively (*p* < 0.001).

**Table 2:** Comparison of selected demographic and anthropometric characteristics between males (*n* = 1,461) and females (*n* = 1,531) in the NHANES (1999–2004).

	Males	Females	*p*
Age (years)	61.2 ± 13.5	61.4 ± 13.5	0.822
Race, %			0.835
Mexican American	28.2	28.9	
Other Hispanic	5.4	5.4	
Non-Hispanic White	38.2	38.1	
Non-Hispanic Black	22.8	23.4	
Other Race – Including Multi-Racial	5.4	4.2	
PIR category, %			<0.001
<1	19.1	27.9	
> = 1	71.9	63.5	
Missing values	9.0	8.6	
Serum cotinine category, %			<0.001
<=0.011	5.9	9.9	
0.011–10	50.7	60.0	
>10	36.5	23.5	
Missing values	7.4	6.6	
BMI category, %			0.001
<25 kg/m^2^	43.5	48.2	
25–30 kg/m^2^	31.5	23.6	
>30 kg/m^2^	22.7	26.0	
Missing values	2.3	2.2	
Physical activity, %			<0.001
None	30.2	34.3	
Moderate	16.7	25.2	
Vigorous	52.9	40.2	
Missing values	0.2	0.3	
Alcohol drink, %			<0.001
No	11.3	24.4	
Yes	57.4	40.6	
Missing values	31.3	35.0	
NHANES cycles			0.371
1999–2000	30.5	32.3	
2001–2002	34.6	35.0	
2003–2004	34.9	32.7	

Mean ± standard deviation. Percentage.

PIR, poverty income ratio. BMI, body mass index. NHANES, National Health and Nutrition Examination Survey.

In the examination of overall death rate, we discovered that there was no notable distinction in the correlation between the five PCDDs and overall death rate in the findings of model 1 (Table 3). In the second model, there was a correlation observed between a rise of 1 log unit in 1,2,3,4,6,7,8,9-octachlorodibenzo-p-dioxin and all-cause mortality (HR = 1.79, 95% CI 1.22, 2.53, *p* = 0.003). There was no significant correlation found between the remaining four PCDDs and all-cause mortality. In our examination of cancer death rates, we discovered that there was no notable distinction in the correlation between the five PCDDs and cancer mortality in model 1. In the second model, it was discovered that a rise of 1 log unit in the presence of 1,2,3,4,6,7,8-heptachlorodibenzofuran led to an increased likelihood of cancer-related deaths (HR = 1.98, 95% CI 1.05, 3.43, *p* = 0.034). The remaining four PCDDs were not statistically associated with cancer mortality.

**Table 3:** The association of polychlorinated dibenzo-p-dioxins (PCDDs) concentration with all-cause mortality, cardiovascular mortality and cancer mortality in NHANES 1999–2004.

	Model 1			Model 2		
PCDDs	HR	95% CI	*p*	HR	95% CI	*p*
1,2,3,6,7,8-Hexachlorodibenzo-p-dioxin						
All mortality	0.99	0.84, 1.17	0.920	1.04	0.85, 1.27	0.725
Cancer mortality	0.73	0.52, 1.02	0.065	0.75	0.50, 1.14	0.178
1,2,3,4,6,7,8-Heptachlororodibenzo-p-dioxin						
All mortality	0.69	0.55, 0.87	0.001	0.45	0.32, 0.64	<0.001
Cancer mortality	0.52	0.32, 0.84	0.008	0.48	0.23, 1.02	0.055
1,2,3,4,6,7,8,9-Octachlorodibenzo-p-dioxin						
All mortality	0.99	0.79, 1.24	0.903	1.76	1.22, 2.53	0.003
Cancer mortality	0.66	0.40, 1.07	0.095	1.08	0.49, 2.36	0.848
1,2,3,4,6,7,8-Heptachlorodibenzofuran						
All mortality	0.97	0.76, 1.23	0.784	1.04	0.78, 1.38	0.776
Cancer mortality	1.16	0.70, 1.92	0.570	1.90	1.05, 3.43	0.034
3,3′,4,4′,5-Pentachlorobiphenyl						
All mortality	0.86	0.72, 1.03	0.093	0.98	0.80, 1.20	0.878
Cancer mortality	0.71	0.49, 1.02	0.067	0.87	0.57, 1.32	0.503

Model 1, age, gender, PIR category, serum cotinine category, alcohol drink status, physical activity status and NHANES cycles.

Model 2, model 1 plus all polychlorinated dibenzo-p-dioxins.

HR, hazard ratio; CI, confidence interval.

Upon analyzing the quartiles of PCDDs, it was determined that there was no heightened risk associated with all-cause mortality for any of the five PCDDs (refer to Table 4). In the findings of the association analysis concerning cancer mortality (Table 5), it was noted that the HR for the uppermost quantile of 1,2,3,4,6,7,8-heptachlorodibenzofuran, in comparison to the lowest quantile, was 2.00 (1.06, 3.79). The HR for the highest quantile of 1,2,3,4,6,7,8-heptachlorodibenzofuran, compared to the lowest quantile, was 2.23 (1.13, 4.40). The other four PCDDs were not statistically associated with cancer mortality risk.

**Table 4:** The association of quartiles polychlorinated dibenzo-p-dioxins concentration with all-cause mortality in NHANES 1999–2004.

PCDDs	Q1	Q2	Q3	Q4
1,2,3,6,7,8-Hexachlorodibenzo-p-dioxin	Ref	1.16 (0.87, 1.53)	0.94 (0.70, 1.25)	1.22 (0.90, 1.66)
1,2,3,4,6,7,8-Heptachlororodibenzo-p-dioxin	Ref	0.79 (0.59, 1.06)	0.64 (0.47, 0.89)	0.55 (0.38, 0.80)
1,2,3,4,6,7,8,9-Octachlorodibenzo-p-dioxin	Ref	1.14 (0.85, 1.53)	1.21 (0.88, 1.66)	1.40 (0.99, 2.00)
1,2,3,4,6,7,8-Heptachlorodibenzofuran	Ref	1.11 (0.85, 1.43)	1.01 (0.76, 1.34)	1.08 (0.80, 1.47)
3,3′,4,4′,5-Pentachlorobiphenyl	Ref	1.09 (0.83, 1.41)	0.87 (0.67, 1.14)	0.88 (0.66, 1.17)

Q, quartile; PCDD, polychlorinated dibenzo-p-dioxin.

Adjusted as age, gender, PIR category, serum cotinine category, alcohol drink status, physical activity status, NHANES cycles, and all polychlorinated dibenzo-p-dioxins.

**Table 5:** The association of quartiles polychlorinated dibenzo-p-dioxins (PCDDs) concentration with cancer mortality in NHANES 1999–2004.

PCDDs	Q1	Q2	Q3	Q4
1,2,3,6,7,8-Hexachlorodibenzo-p-dioxin	Ref	0.86 (0.49, 1.50)	0.79 (0.45, 1.41)	0.87 (0.46, 1.65)
1,2,3,4,6,7,8-Heptachlororodibenzo-p-dioxin	Ref	0.86 (0.48, 1.53)	0.70 (0.37, 1.34)	0.59 (0.28, 1.24)
1,2,3,4,6,7,8,9-Octachlorodibenzo-p-dioxin	Ref	1.18 (0.66, 2.13)	1.06 (0.55, 2.02)	1.51 (0.74, 3.06)
1,2,3,4,6,7,8-Heptachlorodibenzofuran	Ref	1.77 (0.98, 3.19)	2.00 (1.06, 3.79)	2.23 (1.13, 4.40)
3,3′,4,4′,5-Pentachlorobiphenyl	Ref	1.05 (0.61, 1.80)	1.00 (0.58, 1.75)	0.90 (0.50, 1.64)

Q, quartile; PCDD, polychlorinated dibenzo-p-dioxin.

Adjusted as age, gender, PIR category, serum cotinine category, alcohol drink status, physical activity status, NHANES cycles, and all polychlorinated dibenzo-p-dioxins.

**Figure 1:**
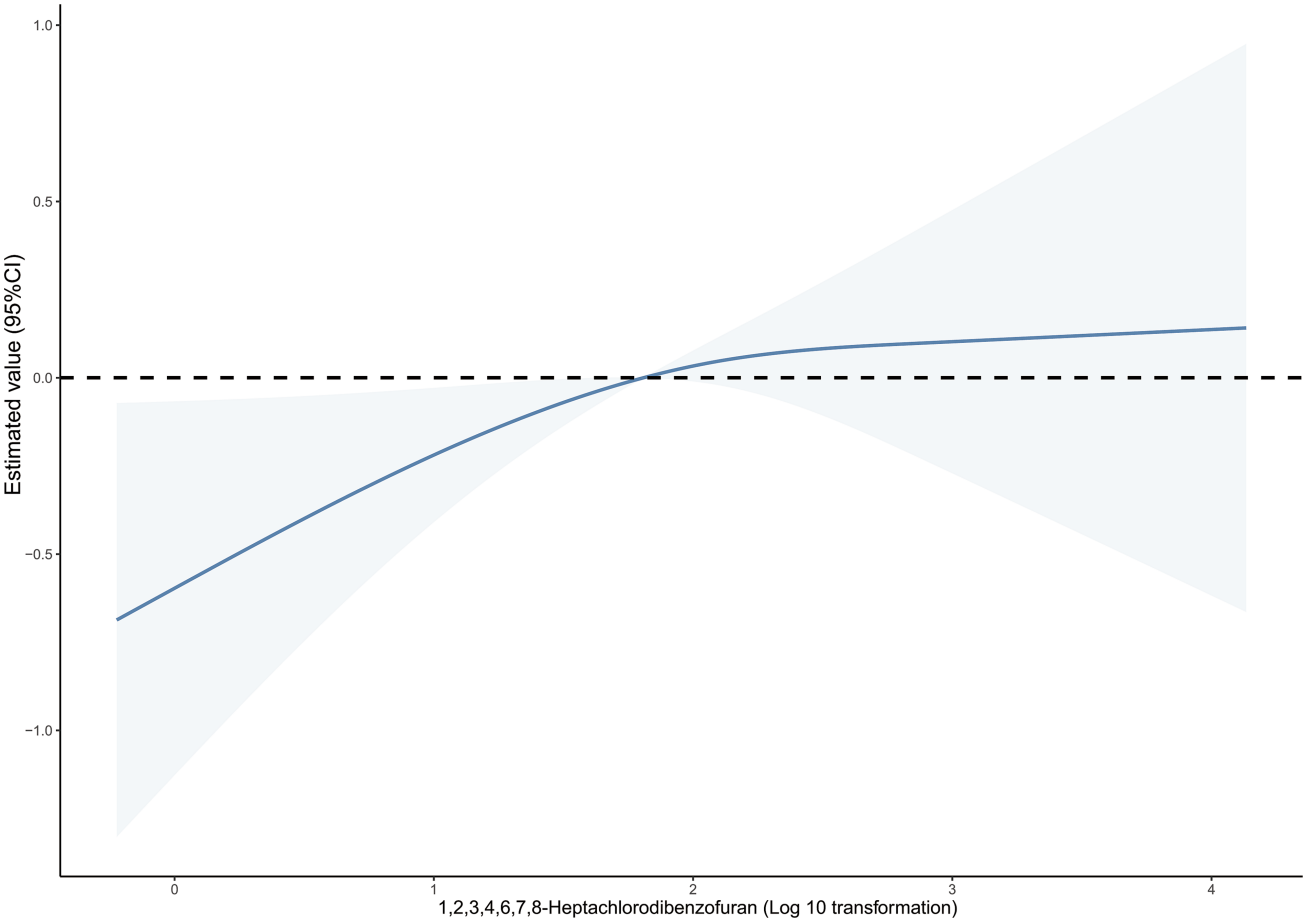
Dose–response relationship between blood 1,2,3,4,6,7,8-Heptachlorodibenzofuran levels and cancer mortality. The solid line and dashed line represent the estimated values and their 95% confidence intervals.

In addition, we examined the correlation between cancer mortality risk and the dosage of 1,2,3,4,6,7,8-heptachlorodibenzofuran using an RCS model (Figure 1). The findings indicated a direct correlation between the compound 1,2,3,4,6,7,8-heptachlorodibenzofuran and the risk of cancer-related deaths, with a P_linear_ value of 0.044. The above two variables did not exhibit a nonlinear relationship, with a P_non-linear_ value of 0.262. Furthermore, we carried out a sensitivity analysis as well. By either eliminating the covariate PIR variable or enhancing the covariate education level, the findings remained consistent, indicating the robustness of our results (Tables 6, 7).

**Table 6:** The association of quartiles polychlorinated dibenzo-p-dioxins (PCDDs) concentration with cancer mortality in NHANES 1999–2004.

PCDDs	Q1	Q2	Q3	Q4
1,2,3,6,7,8-Hexachlorodibenzo-p-dioxin	Ref	0.79 (0.46, 1.37)	0.72 (0.41, 1.28)	0.79 (0.43, 1.48)
1,2,3,4,6,7,8-Heptachlororodibenzo-p-dioxin	Ref	0.88 (0.49, 1.56)	0.82 (0.42, 1.59)	0.72 (0.33, 1.55)
1,2,3,4,6,7,8,9-Octachlorodibenzo-p-dioxin	Ref	1.09 (0.61, 1.95)	0.99 (0.52, 1.88)	1.51 (0.74, 3.06)
1,2,3,4,6,7,8-Heptachlorodibenzofuran	Ref	1.63 (0.91, 2.92)	1.83 (0.98, 3.45)	2.02 (1.03, 3.96)
3,3′,4,4′,5-Pentachlorobiphenyl	Ref	0.99 (0.58, 1.69)	1.00 (0.58, 1.75)	0.93 (0.51, 1.69)

Adjusted as age, gender, serum cotinine category, alcohol drink status, physical activity status, NHANES cycles, and all polychlorinated dibenzo-p-dioxins.

**Table 7:** The association of quartiles polychlorinated dibenzo-p-dioxins (PCDDs) concentration with cancer mortality in NHANES 1999–2004.

PCDDs	Q1	Q2	Q3	Q4
1,2,3,6,7,8-Hexachlorodibenzo-p-dioxin	Ref	0.81 (0.47, 1.40)	0.76 (0.43, 1.34)	0.81 (0.43, 1.52)
1,2,3,4,6,7,8-Heptachlororodibenzo-p-dioxin	Ref	0.90 (0.50, 1.60)	0.83 (0.43, 1.62)	0.70 (0.33, 1.52)
1,2,3,4,6,7,8,9-Octachlorodibenzo-p-dioxin	Ref	1.11 (0.62, 2.00)	1.01 (0.53, 1.94)	1.39 (0.68, 2.84)
1,2,3,4,6,7,8-Heptachlorodibenzofuran	Ref	1.66 (0.92, 2.99)	1.88 (1.00, 3.55)	2.03 (1.03, 4.01)
3,3′,4,4′,5-Pentachlorobiphenyl	Ref	0.98 (0.57, 1.68)	1.02 (0.59, 1.78)	0.93 (0.51, 1.70)

Adjusted as age, gender, education levels, PIR category, serum cotinine category, alcohol drink status, physical activity status, NHANES cycles, and all polychlorinated dibenzo-p-dioxins.

## Discussion

The findings of the current research revealed that higher levels of exposure to 1,2,3,4,6,7,8-heptachlorodibenzofuran, a form of PCDD, were linked to an elevated likelihood of cancer fatality. The risk of cancer mortality increased by 90% with the addition of each extra 1,2,3,4,6,7,8-heptachlorodibenzofuran. In addition, 1,2,3,4,6,7,8-heptachlorodibenzofuran was linearly associated with cancer mortality.

In the general population, our research discovered a correlation between exposure to PCDD and the likelihood of cancer-related deaths. In previous studies, the cancer mortality of environmental chemicals in occupational populations has been widely studied. Occupationally exposed individuals have a higher concentration of chemicals in their bodies compared to the general population. According to Leroyer et al.’s study (13), there was no observed link between cancer mortality and the exposure to various chemicals in the workplace. In the study conducted by Zhivin and colleagues (14), the mortality risk of kidney and bladder cancer due to exposure to hydrazine and other fuels in the population exposed to these substances at work was observed, however, no statistically significant distinction was found. The findings from these occupational exposure categories did not establish a connection between environmental chemicals and the risk of death, indicating that not all occupational exposures to environmental chemicals are linked to mortality risk. There is chemical specificity between environmental chemicals and mortality risk. An elevated amount of dioxin exposure is linked to a rise in mortality rates from bladder cancer (15). Furthermore, a meta-analysis indicated that the exposure to dioxin was linked to a heightened likelihood of cancer fatality (16). Furthermore, being exposed to PCDDs in the workplace can lead to a rise in cancer-related deaths (7, 17). These findings additionally demonstrate that there exists a distinct chemical specificity between environmental substances and the risk of mortality. Previous research in the field of mortality risk among the overall populace has indicated a strong correlation between the element cadmium and tumor fatality (18, 19). It is suggested that PCDD may also have similar risks. In contrast, Lin et al. discovered that PCDD did not have any connection with the likelihood of tumor death among the overall populace (11). Upon enlarging the sample size and extending the duration of follow-up, we discovered a correlation between PCDD and a heightened likelihood of tumor fatality. Different results may be discovered with a substantial sample size and an extended duration of follow-up, as recommended.

This study found that nonoccupational exposure to PCDD can cause an increase in tumor mortality, which may have certain public health significance. Currently, numerous elements contribute to the heightened likelihood of tumor fatality, encompassing cadmium of metallic nature (20), PM_2.5_ particles (21), and phthalate substances (22). Nevertheless, these factors alone are insufficient to account for tumor mortality, implying that unidentified environmental substances impact tumor mortality. The findings of our research indicate that even a small amount of PCDD can lead to a higher likelihood of tumor-related deaths, making it the first study to establish this connection. Reducing the exposure to PCDD may potentially decrease the risk of tumor mortality. PCDD can be found in various sources that humans are exposed to, such as emissions from industries, outdated pesticide supplies, heating in households, recycling of electronic waste, and the incineration and combustion of household waste (23). To decrease human PCDD exposure, another approach is to minimize the origin of PCDD exposure. Hence, minimizing the contact with chemicals might lead to a decrease in the presence of chemicals within the body. Hence, considering public health, implementing drug intervention and minimizing chemical contact are two approaches to decrease the level of chemicals in the body, which could potentially lower the chances of tumor fatality among the population.

This study has the following advantages. In the initial investigation using the cohort study approach, we discovered, for the very first instance, that among individuals not occupationally exposed, there exists a direct association between combined exposure to PCDDs and tumor mortality. Furthermore, we observed a noteworthy 90% rise in the likelihood of risk. Furthermore, our research revealed a strong correlation between tumor mortality and the presence of 1,2,3,4,6,7,8-heptachlorodibenzofuran in PCDD. This study also has limitations. Firstly, in this study, the origin of PCDD exposure cannot be disclosed initially. Clarifying the exposure source of PCDD can reduce its harm. Secondly, the PCDD exposure level in this study was based on a single determination, which could introduce bias. Therefore, it is necessary to conduct multiple determinations in future studies to establish a reliable and consistent exposure level. Thirdly, varying individuals may possess distinct PCDD metabolism, potentially resulting in diverse health consequences stemming from identical levels of PCDD exposure. This requires adding variables affecting the metabolic level of PCDD in subsequent studies to reduce such bias. Fourthly, it remains uncertain if the findings of this research can be universally applied worldwide. People in different regions have different exposure levels to PCDDs and different life spans. Hence, additional areas and larger sample sizes should be examined. Fifthly, occupational exposure is one of the important reasons affecting PCDD, but due to the lack of occupational information data in NHANES, we can not adjust this variable in the statistical analysis of the present study. This may affect our results, which needs attention in the follow-up study. Sixthly, this study was conducted by sampling, and there was also sampling bias existing. In the discussion part, we gave the biological evidence of potential PCDD causing tumor, which may reduce such bias, but the sampling bias should not be ignored in the future study. Seventhly, previous studies have found that PCDD residues can be partially detected in eggs, mussels, milk and cheese produced around the areas where PCDD industry comes from (24–27). Therefore, the intake of eggs, mussels, milk and cheese produced may be a confounding factor of PCDD, which needs to be collected as a covariate in subsequent studies. Eighthly, dietary exposure is the main source of non occupational exposure to human to exposure to PCDD. Mussels, as a kind of seafood, and PCDD is accumulated in it (25). Eating seafood can reduce the total cause of death, although there is no statistical difference (28). These evidences suggest that the dietary exposure, such as consumption of mussels, may lead to biased results.

## Conclusion

This research revealed that PCDD may elevate the likelihood of cancer-related deaths among the overall populace.

## Data availability statement

The original contributions presented in the study are included in the article/supplementary materials, further inquiries can be directed to the corresponding author/s.

## Ethics statement

The consent form was signed by the survey participants, and the participants consented to storing specimens of their blood for future research. The CDC/NCHS Ethics Review Board approved the NHANES study and gave approval for public dissemination.

## Author contributions

LZ: Writing – original draft. XZ: Data curation, Writing – review & editing. ZG: Formal analysis, Writing – original draft. CZ: Validation, Writing – review & editing. DQ: Methodology, Writing – review & editing. QY: Conceptualization, Supervision, Writing – original draft.

## Footnotes

^1^ https://wwwn.cdc.gov/nchs/nhanes/ResponseRates.aspx
